# Expansion and diversification of the *SET *domain gene family following whole-genome duplications in *Populus trichocarpa*

**DOI:** 10.1186/1471-2148-12-51

**Published:** 2012-04-12

**Authors:** Li Lei, Shi-Liang Zhou, Hong Ma, Liang-Sheng Zhang

**Affiliations:** 1 State Key Laboratory of Systematic and Evolutionary Botany, Institute of Botany, the Chinese Academy of Sciences, Beijing 100093, China; 2 State Key Laboratory of Genetic Engineering, Institute of Plants Biology, Center for Evolutionary Biology, School of Life Sciences, Fudan University, Shanghai 200433, China; 3 Graduate University of the Chinese Academy of Sciences, Beijing 100039, China

## Abstract

**Background:**

Histone lysine methylation modifies chromatin structure and regulates eukaryotic gene transcription and a variety of developmental and physiological processes. SET domain proteins are lysine methyltransferases containing the evolutionarily-conserved SET domain, which is known to be the catalytic domain.

**Results:**

We identified 59 *SET *genes in the *Populus *genome. Phylogenetic analyses of 106 *SET *genes from *Populus *and *Arabidopsis *supported the clustering of *SET *genes into six distinct subfamilies and identified 19 duplicated gene pairs in *Populus*. The chromosome locations of these gene pairs and the distribution of synonymous substitution rates showed that the expansion of the *SET *gene family might be caused by large-scale duplications in *Populus*. Comparison of gene structures and domain architectures of each duplicate pair indicated that divergence took place at the 3'- and 5'-terminal transcribed regions and at the N- and C-termini of the predicted proteins, respectively. Expression profile analysis of *Populus SET *genes suggested that most *Populus SET *genes were expressed widely, many with the highest expression in young leaves. In particular, the expression profiles of 12 of the 19 duplicated gene pairs fell into two types of expression patterns.

**Conclusions:**

The 19 duplicated *SET *genes could have originated from whole genome duplication events. The differences in *SET *gene structure, domain architecture, and expression profiles in various tissues of *Populus *suggest that members of the *SET *gene family have a variety of developmental and physiological functions. Our study provides clues about the evolution of epigenetic regulation of chromatin structure and gene expression.

## Background

Gene expression is regulated by many factors acting in concert with the status of the chromatin environment, particularly histone methylation [1]. The SET-domain-containing protein family is a major player in histone methylation. These proteins are responsible for the methylation of lysine (K) residues in various histones, specifically K4, K9, K27, and K36 in histone H3 and K20 in histone H4 [2]; H3K79 is an exception [3]. All members of this family share a highly conserved SET domain [2], named for three *Drosophila melanogaster *proteins: ***S**uppressor of variegation 3-9 (Su(var)3-9) *(Suv), ***E**nhancer of Zeste *(E(z)) and ***T**rithorax *(Trx). This domain has approximately 130 amino acids and has been found in all eukaryotic organisms studied so far [2]. Proteins containing the SET domain can also be found in viruses as well as both domains of prokaryotes [4,5].

Recent studies have revealed that the *SET *domain (here after referred as *SET*) genes are important for regulating growth and reproduction processes, such as control of flowering time and embryogenesis in plants [6]. Genome sequencing has uncovered many genes encoding SET-domain proteins; in particular, *Arabidopsis SET *genes are the best annotated and characterized. For example, the *Arabidopsis CURLY LEAF *(*CLF*) gene is required for stable repression of the floral homeotic gene *AGAMOUS *in leaves and stems [7]. *ARABIDOPSIS TRITHORAX 1 *(*ATX1*) functions as an activator of homeotic genes, like *Trithorax *in animal systems [8]. *ARABIDOPSIS *TRITHORAX-RELATED PROTEIN 7 (ATXR7) is an H3K4 methylase required for proper expression of the *Flowering Locus C *(*FLC*) gene [9]. The *Arabidopsis *ASH1 HOMOLOG 2 (ASHH2) protein has been suggested to methylate H3K4 and/or H3K36, similar to *Drosophila *ASH1 and yeast SET2, a H3K36 histone methyltransferase (HMT) [10]. Other *SET *genes are associated with embryogenesis, including *MEDEA *(*MEA*); a maternally inherited loss-of-function *mea *allele results in embryo abortion and prolonged endosperm production [11]. Recently, *ATXR3 *is crucial for both sporophyte and gametophyte development and encodes the major enzyme responsible for trimethylation of H3K4 [12,13].

In plants, at least 47, 33, 31, and 43 *SET *genes have been identified in *Arabidopsis*, grape, maize and rice, respectively [14-16]. In both *Arabidopsis *and rice it has been determined that many *SET *genes are located in large blocks of related regions derived from whole genome duplication events, indicating that whole genome duplication could be an important contributor to the duplication of *SET *genes [15]. In addition, different classifications of *SET *genes were used in different plants. Initially, 37 putative *Arabidopsis SET *domain proteins were classified into four distinct classes: (I) enhancer of zeste [E(z)] homologs; (II) trithorax (Trx) homologs and related proteins; (III) Ash1 homologs and related proteins; and (IV) suppressor of variegation [Su(var)] homologs and related proteins [17]. In another study, 32 *Arabidopsis *and 22 maize *SET *genes were classified into five classes according to phylogenetic relationships and domain organization [18]. More recently, two additional classes (VI and VII) were recognized for *SET *genes in *Arabidopsis*, grape, maize, and rice [14-16]. Interestingly, in *Arabidopsis *several genes in class III, like *ATXR3*, were shown to be crucial for both sporophyte and gametophyte development [12,13]. Moreover, *Arabidopsis *has ten Su(var) homologue (*SUVH*) genes belonging to Class V, including several that control heterochromatic domains. Loss of function of these genes suppresses gene silencing, whereas overexpression enhances silencing, causing ectopic heterochromatization and significant growth defects in *Arabidopsis *[19]. Therefore, *SET *genes in different subfamilies could have diverse functions.

Previous studies of *SET *genes have focused on annotation and *Arabidopsis *functional characterizations [7-13,19]; in addition evolutionary analyses have been limited to herbaceous plants [14-18]. Trees are distinct from herbaceous species in many ways: they have a self-supporting structure, the secondary growth or wood, and a much longer lifespan [20,21]. The regulatory networks and molecular mechanisms that underlie these unique properties cannot be investigated through the examination of nontree species. Therefore, it is worthwhile to study *SET *genes in trees, thereby improving our understanding in their functions and the evolution of *SET *genes. The recently completed genome sequence of the model tree, *Populus trichocarpa *(hereafter called *Populus*) [22], provides a great opportunity to investigate these issues.

Molecular evidence suggests that *Arabidopsis *and *Populus *shared their last common ancestor as much as 100 to 120 million years ago [22]. Since then, *Arabidopsis *and *Populus *have evolved different life histories, including herbaceous versus arboreal development, annual versus perennial habit, and self-pollination versus cross-pollination strategies [20,21]. In addition, since they diverged from each other, *Populus *has experienced whole genome duplication once, whereas *Arabidopsis *has twice [22,23]. In plants, evolutionary diversity has been hypothesized to be modulated directly or indirectly by epigenetic regulations [24]. Therefore, the *SET *gene family, among the most important epigenetic regulators, could be postulated to contribute substantially to the evolutionary innovations in plant diversity.

We conducted a comparative analysis of *SET *genes from *Arabidopsis *and *Populus *to address the key question: how have *SET *genes evolved in *Populus *after the divergence of *Arabidopsis *and *Populus*. In particular, how did the *SET *gene family expand and diversify in *Populus*? In this study, we performed comprehensive analyses of *SET *genes from *Populus*, including phylogeny, gene structure, domain architecture, gene duplication and diversification, and expression profiling analyses. Our results provide insight into the function of *Populus SET *genes and provide a basis for understanding how gene functions, particularly functions involved in the development of trees, have evolved.

## Results

### Identification of *SET *genes in *Populus*

We obtained the *Populus *whole-genome shotgun trace data from JGI and identified all predicted proteins containing SET domains. We named *Populus SET *genes based on the previously reported *Arabidopsis SET *gene names [17] and phylogenetic relationships here (Table 1). The *Arabidopsis SET *genes follow the standard gene symbol conventions with all capital letters; for genes from *Populus*, the first letter was capitalized but others were in lower case, using the same name as the closest *Arabidopsis *homolog. If the name of the closest *Arabidopsis *homolog is already used for a *Populus *gene, then the name of the next closest homolog is used; if two or more *Populus *genes are equally close to a single *Arabidopsis *gene, then the same name is used followed by the letters "a", "b", etc. A total of 59 *Populus SET *genes were identified and compared to *SET *genes in *Arabidopsis *(47) and rice (43) [15]. There were a total of 45,555 protein-coding genes in *Populus*, 27,417 in *Arabidopsis *and 56,662 in rice. This suggested that the numbers of *SET *genes were not proportional to the sizes of the predicted gene sets.

**Table 1:** Members of the *SET *gene family in *Populus*

Gene name	Protein length	subfamily*	ESTs	Gene position	Gene Model
PoSuvh2	453	Suv	-	LG_VIII(11654980-11656338)	gw1.VIII.652.1

PoSuvh9	519	Suv	6	LG_X(5498008-5499564)	gw1.X.4649.1

PoSuvh5	513	Suv	-	LG_III(9558215-9561181)	gw1.118.279.1

PoSuvh4a	486	Suv	-	LG_III(14574409-14583315)	gw1.III.353.1

PoSuvh4b	525	Suv	8	LG_II(22271452-22288911)	gw1.II.373.1

PoSuvh4c	509	Suv	-	LG_XIV(7067483-7077824)	gw1.XIV.3033.1

PoSuvh3	496	Suv	-	LG_VI(1774010-1775496)	gw1.VI.98.1

PoSuvh10	496	Suv	7	LG_XVI(1303523-1305010)	gw1.XVI.1034.1

PoSuvh1	653	Suv	2	LG_III(17984592-17986613)	fgenesh4_pg.C_LG_III001771

PoSuvh7	512	Suv	7	LG_I(1436489-1438607)	estExt_Genewise1_v1.C_LG_I6844

PoSuvr5a	1428	Suv	-	LG_V(4008809-4012099)	gw1.V.3747.1

PoSuvr5b	1517	Suv	5	LG_VII(10134577-10137249)	estExt_Genewise1_v1.C_LG_VII3275

PoSuvr1	296	Suv	-	LG_IV(16108605-16109961)	gw1.IV.1292.1

PoSuvr2	414	Suv	-	LG_IX(1847452-1849907)	gw1.IX.1069.1

PoSuvr4	401	Suv	3	LG_XIII(3470303-3478528)	gw1.XIII.1724.1

PoSuvr4a	714	Suv	2	LG_XIV(12538712-12542929)	gw1.XIV.76.1

PoSuvr4b	464	Suv	4	LG_II(23795948-23799204)	gw1.II.215.1

PoSuvr3	340	Suv	5	LG_XIII(4848844-4850695)	gw1.XIII.2167.1

PoAtxr6a	319	Atxr5	-	LG_XII(2169730-2172278)	gw1.XII.888.1

PoAtxr6b	325	Atxr5	-	LG_XV(668228-670843)	gw1.XV.292.1

PoAtxr5a	304	Atxr5	9	LG_VII(2505750-2509040)	fgenesh4_pm.C_LG_VII000124

PoAtxr5b	333	Atxr5	1	LG_V(3899727-3900751)	gw1.70.74.1

PoAshh3	351	Ash	8	LG_XVII(191202-195604)	eugene3.01700004

PoAshh4	281	Ash	7	LG_VII(13836-18843)	eugene3.00070002

PoAshh1	495	Ash	8	LG_V(13318562-13324389)	fgenesh4_pm.C_LG_V000437

PoAshr3	402	Ash	-	LG_XVIII(10390889-10400394)	gw1.XVIII.3091.1

PoAshh2a	605	Ash	1	LG_II(5451879-5460030)	eugene3.00020732

PoAshh2b	594	Ash	-	LG_V(1177943-12003046)	gw1.V.27.1

PoAtxr3a	2350	Atxr3	7	LG_VII(1678369-1689434)	fgenesh4_pg.C_LG_VII000266

PoAtxr3b	2476	Atxr3	28	LG_XVII(5241549-5229352)	eugene3.00640010

PoSwna	852	E(z)	10	LG_II(16109075-16116062)	gw1.II.890.1

PoSwnb	812	E(z)	1	LG_XIV(5213392-5220923)	estExt_Genewise1_v1.C_LG_XIV2284

PoClfa	917	E(z)	5	LG_V(5117508-5125370)	estExt_fgenesh4_pg.C_LG_V0535

PoClfb	892	E(z)	1	LG_VII(8674891-8681980)	estExt_Genewise1_v1.C_LG_VII2923

PoAtxr7a	1390	Trx	2	LG_II(69963-76718)	fgenesh4_pg.C_LG_II000009

PoAtxr7b	1149	Trx	3	LG_V(25489363-25495946)	POPTR_0005s28130

PoAtx6	712	Trx	-	LG_XVIII(1929427-1923848)	POPTR_0018s02170

PoAtx1	1014	Trx	6	LG_II(20140580-20153598)	fgenesh4_pg.C_LG_II002136

PoAtx2	1050	Trx	5	LG_XIV(7247301-7259403)	gw1.XIV.3109.1

PoAtx3a	667	Trx	2	LG_II(13105976-13113638)	fgenesh4_pg.C_LG_II001571

PoAtx3b	908	Trx	6	LG_XIV(3468877-3477546)	gw1.XIV.1697.1

PoAtx5	1070	Trx	3	LG_XV(992498-1001204)	gw1.XV.426.1

PoAtx4	1078	Trx	4	LG_XII(1824820-1833670)	gw1.XII.196.1

PoAshr2	326	SMYD	3	LG_I(5175894-5176871)	gw1.I.8071.1

PoAtxr4	283	SMYD	7	LG_VI(5075142-10640133)	gw1.VI.14.1

PoAtxr1	542	SMYD	-	LG_VIII(5563751-5563753)	fgenesh4_pg.C_LG_VIII000780

PoAtxr2	398	SMYD	9	LG_XVII(5018556-5014447)	estExt_fgenesh4_pm.C_640013

PoAshr1	458	SMYD	4	LG_V(11020007-11025452)	eugene3.00050808

PoSetd8	167	SETD	4	LG_III(13995189-13996722)	gw1.III.385.1

PoSetd3	362	SETD	1	LG_VIII(8841653-8843777)	fgenesh4_pg.C_LG_VIII001195

PoSetd4	513	SETD	2	LG_VI(16825966-16820213)	POPTR_0006s18410

PoSetd7	468	SETD	2	LG_IV(11613451-11616746)	gw1.IV.4590.1

PoSetd10	444	SETD	12	LG_IV(7403229-7405799)	gw1.IV.3768.1

PoSetd6	439	SETD	1	LG_VIII(2548612-2554748)	gw1.VIII.1187.1

PoSetd9	502	SETD	9	LG_VII(9438694-9433649)	eugene3.01730002

PoSetd5	503	SETD	12	LG_XIV(9526856-9530848)	eugene3.00141165

PoSetd1	551	SETD	4	LG_XIV(2038778-2044599)	gw1.XIV.1045.1

PoSetd2a	490	SETD	7	LG_VIII(5640809-5644314)	gw1.VIII.2559.1

PoSetd2b	345	SETD	-	LG_X(15047671-15051672)	gw1.X.1011.1

**Figure 1:**
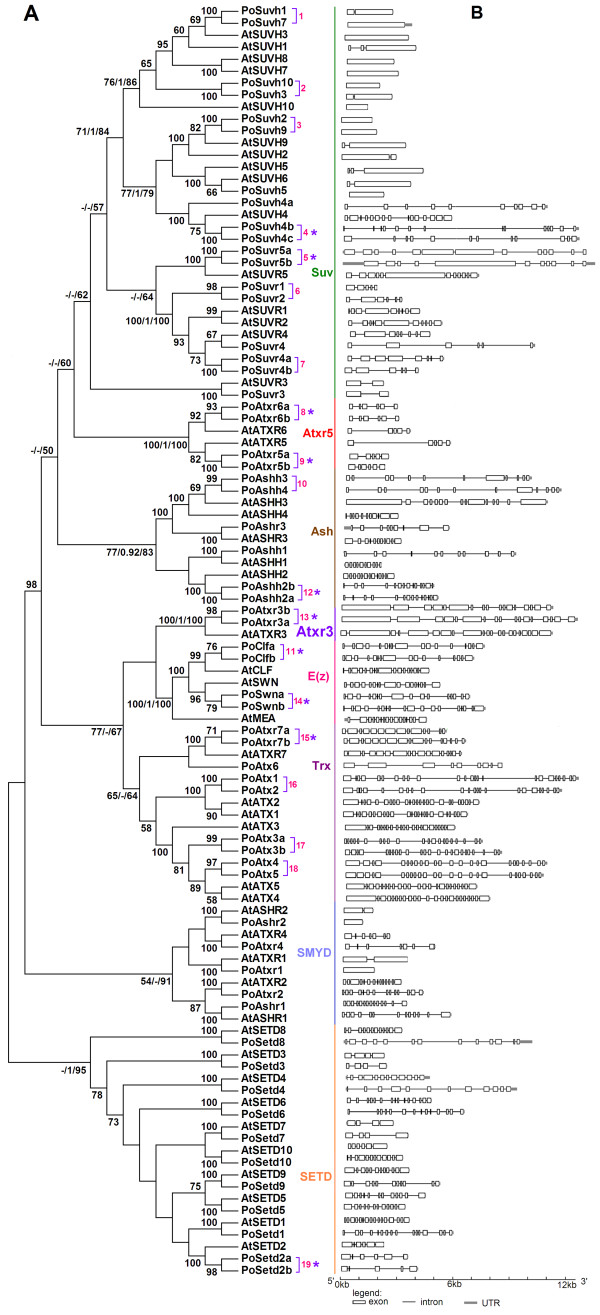
**A phylogenetic tree and a schematic diagram for intron/exon structures of *SET *genes in *Populus *and *Arabidopsis***. **(A) **Unrooted tree constructed with NJ methods using SET domain amino acid sequences from *Populus *and *Arabidopsis SET *proteins. ML bootstrap values, Posterior probabilities from BI and NJ bootstrap values analyses are presented for each main clade. ML and NJ bootstrap values above 50%, and BI posterior probabilities greater than 0.90 analyses are shown. "-" represents the NJ or ML bootstrap values below 50 or the posterior probabilities low 0.90. There are eight subfamilies. The pink brackets followed by numbers denote duplicated gene pairs in *Populus*. **(B) **The intron-exon structure of the SET gene family in *Populus *and *Arabidopsis*.

* Represent the subfamilies shown in Figure 1

### Phylogeny and gene structures of *Populus SET *genes

To understand the evolution of *Populus SET *genes, we performed unrooted phylogenetic analyses on the 106 *SET *genes from *Populus *(59 genes) and *Arabidopsis *(47) using Maximum Likelihood (ML), Bayesian inference (BI) and Neighbour-Joining (NJ) methods (Figures 1A and Additional file 1). The tree topologies produced by the three methods are largely consistent, with only minor differences at interior nodes (Additional file 1). The NJ tree is shown in Figure 1A and discussed below. Most *Populus SET *genes clustered with their homologs in *Arabidopsis*. Sometimes, however, two *Populus *genes clustered together with either a single *Arabidopsis *gene, or without any corresponding gene in *Arabidopsis *(Figure 1A, pink single brackets followed by numbers). In total, there are 19 such pairs of *Populus SET *genes.

The phylogenetic tree topology and the predicted protein domain compositions also allow the grouping of the 106 *SET *genes in *Arabidopsis *and *Populus *into eight subfamilies (named Suv, Atxr5, Ash, Atxr3, E(z), Trx, SMYD and SETD; Figure 1A), generally in accordance with those in other plants [14,16-18]. Nevertheless, there were some differences from prior classifications [16-18]. One notable difference is that recent classifications placed *MEA, CLF *and *SWN *in class I and *ATX1, ATX2, ATX3, ATX4, ATX5, ATXR3 and ATXR7 *in Class III [14-16], but our results had all of these genes clustering together, forming a monophyletic clade with bootstrap support of 77% (Figure 1A). In addition, each subfamily formed a group with high bootstrap support in the unrooted ML/BI/NJ analysis and may have the same ancestral origin.

In general, members from the same subfamily shared similar exon/intron structures, e.g. intron number and exon length; however, some members of the Suv, SMYD and SETD subfamilies (Figure 1B) had structural differences from other members. In the Suv subfamily, retrotransposition events have been reported [17,18], which could have contributed to the diversity of the subfamily members. For subfamilies SMYD and SETD, we observed considerable diversity in gene structure and highly divergent sequences among subfamily members, but this diversity and its possible functions have rarely been reported.

### Expansion and evolution of the *SET *gene family in *Populus*

**Figure 2:**
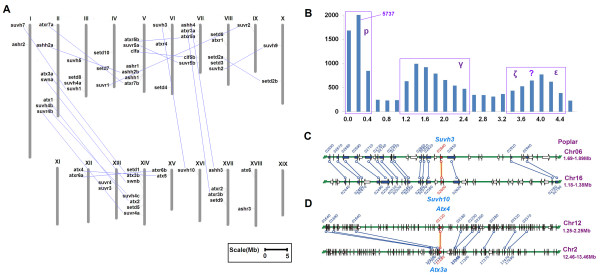
**Positions of *SET *genes on *Populus *chromosomes**. **(A) **Map of the *SET *gene positions on *Populus *chromosomes. Numbers refer to locus ID as listed in Table 1. The *SET *genes found on duplicated chromosomal segments are connected by lines. **(B) **Frequency distribution of *K_s _*between all syntenically duplicated gene pairs in *Populus*. The light blue box indicates a recent polyploidy event (since the split from the *Arabidopsis *lineage), denoted p; the pink box marks a duplication event apparently shared among the eurosids (γ); the dark blue box denotes a possible overlap of ancient polyploidy events shared with all the seeds plants (ε) or angiosperms (ζ). **(C) **One example of the detailed locations of representative pairs of genes duplicated in recent polyploidy events in the syntenic region. **(D) **Detailed location of representative duplicated pairs from ancient polyploidy events in the syntenic region.

The phylogenetic analysis of *Populus SET *genes indicates that they have experienced multiple gene duplication events. Gene duplication mechanisms include tandem duplication and large segmental/whole-genome duplication (WGD). To examine the relative contribution of each of these mechanisms in the expansion of the *SET *gene family, each member was electronically mapped to loci across all 16 *Populus *chromosomes and 4 additional scaffolds according to the location information provided by the JGI database [22] (Figure 2A). Chromosomes LG XI and LG XIX did not contain any *SET *genes (Figure 2A). The highest number of *SET *genes (7, or 11.9% of the total) was found on chromosome II, V and XIV (Figure 2A). Intriguingly, we did not identify gene clusters on any *Populus *chromosomes (Figure 2A), indicating that tandem duplication was not the cause of the detected duplicates in the *Populus SET *gene family. This is similar to the lack of tandem duplication events in the *Arabidopsis *and rice *SET *gene families [15].

Previous studies showed that the *Populus *genome contains evidence for three or more distinct WGD events [22,25]. Actually, strong support has been reported for two ancient WGD events in ancestral lineages shortly before the diversification of extant seed plants (255-400 Mya, million years ago) and extant angiosperms (150-275 Mya), respectively [25]. We analysed all of the duplicated gene pairs with intragenome syntenic relationships in the *Populus *genome from Plant Genome Duplication Database (PGDD) [23,26]. All 19 of the duplicated pairs of *Populus SET *genes resided in *Populus *segmental duplicated blocks (Figure 2A, gene pairs connected with lines; Table 2 and Additional file 2). Particularly, the *Atx4/Atx5* and *Atx3a/Atx3b* pairs were also located on two pairs of duplicated segmental blocks (Figure 2D and Additional file 2).

**Table 2:** Estimated dates of the duplication events leading to pairs of *SET *genes in *Populus *and *Arabidopsis*

Species	Duplicated pair		Number of anchors	Mean *K_s_*	SD *K_s_*	Estimated time (Mya)
*Populus*	Suvh2	Suvh9	777	0.2674	0.0922	14.69

	Suvh3	Suvh10	84	0.2841	0.1030	15.61

	Suvh4b	Suvh4c	14	0.2566	0.0642	14.10

	Suvh7	Suvh1	105	0.2597	0.0715	14.27

	Suvr1	Suvr2	176	0.283	0.0956	15.55

	Suvr4a	Suvr4b	15	0.2898	0.0978	15.93

	Suvr5a	Suvr5b	19	0.2686	0.0968	14.76

	Atxr5a	Atxr5b	118	0.2737	0.0927	15.04

	Atxr6a	Atxr6b	46	0.2657	0.0875	14.60

	Ashh2a	Ashh2b	381	0.2683	0.0894	14.74

	Ashh4	Ashh3	16	0.2568	0.0723	14.11

	Atxr3a	Atxr3b	109	0.2758	0.0877	15.15

	Clfa	Clfb	29	0.2844	0.0966	15.62

	Swna	Swnb	394	0.2697	0.0842	14.82

	Atx1	Atx2	11	0.2483	0.0665	13.64

	Atx3a	Atx3b	394	0.2697	0.0842	14.82

	Atx4	Atx5	46	0.2657	0.0875	14.60

	Atxr7a	Atxr7b	566	0.2517	0.0548	13.83

	Setd2a	Setd2b	1034	0.2594	0.0583	14.09

	Atx4/5	Atx3a/b	46	1.5129	0.1836	83.13

*Arabidopsis*	SUVH3	SUVH7	177	0.8413	0.2201	28.04

	SUVR2	SUVR1	33	0.8419	0.2180	28.06

	SWN	MEA	41	0.8182	0.1871	27.27

	ATX1	ATX2	113	0.8635	0.2162	28.78

	ASHH4	ASHH3	138	0.8422	0.2319	28.07

The mean *K_s _*value was calculated for each pair of protein-coding genes within a duplicated block and used to date the duplication events. *K_s _*values of all duplicated gene pairs greater than 1.5 and less than 0.5 were discarded for *Arabidopsis*; *K_s _*greater than 0.4 for *Populus *was also discarded, except Atx4/5 and Atx3a/b

In addition, we calculated the values of synonymous substitutions per synonymous site (*K_s_*) of duplicated gene pairs from PGDD, which we assumed to be correlated with the time of divergence since the genome duplication (Figure 2B). Apparently, the *Populus *duplicates found in syntenic blocks matched with two WGDs (the first and second peaks, denoted *p *and γ previously [23]). There was also a third peak (*K_s_*, 3.6-4.2, denoted ε or ζ), which might be due to the ancient angiosperm-wide and/or seed plant-wide WGDs [25], with possible blurring of the distinction between the two expected peaks due to subsequent *K_s _*rate variation. To estimate the divergence time of the 19 duplicated gene pairs, we calculated their syntenic *K_s _*values. They could be classified at least into two categories (Table 2). The first category included the 19 gene pairs, whose locations in the syntenic region are shown in Figure 2C and Additional file 2. They had an average *K_s _*value between 0.2483-0.2898, corresponding to the first peak (*p*) in Figure 2B. This duplication event was dated to 13.64-15.93 Mya, corresponding to a recent segmental duplication/WGD event in *Populus*, approximately 10-20 Mya after the split from the lineage leading to *Arabidopsis *[22]. The other category included one pair of duplicated blocks (between *Atx4/Atx5* and *Atx3a/Atx3b*); their detailed locations in the syntenic region are shown in Figure 2D and Additional file 2. Interestingly, they had much higher average *K_s _*value, 1.5129 (Table 2), corresponding to the second peak. These duplicated blocks could be due to the retention of genes from an ancient WGD event(s) shared by core eudicots [23]. In addition the *K_s _*values of between *Suvh4a *and *Suvh4b *or *Suvh4c *were 1.6609 or 1.5428, respectively (Additional file 3: Table S1), suggesting that the duplication of Suvh4a and Suvh4b/c could be due to the WGD shared by the core eudicots. Other pairs of poplar *SET *genes have *K_s _*values ranging from 3.6 and 4.6, consistent with them being from even older WGD duplications, but the *K_s _*values are not reliable to assign the specific WGD. Our analysis strong suggests that segmental duplication events, especially those resulting from recent polyploidy events, have contributed to the expansion of the *SET *domain gene family in *Populus*.

### Domain diversity in *Populus SET *gene family

**Figure 3:**
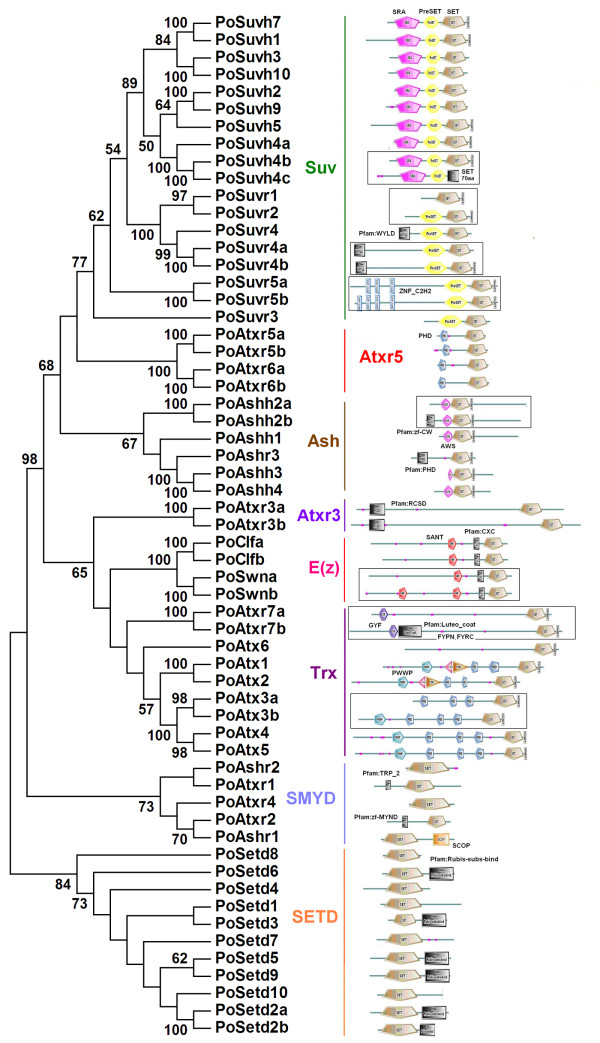
**An unrooted NJ tree using SET domain amino acid sequences from *Populus SET *genes (A) and the domain architecture of SET proteins (B)**. The tree depicts eight subfamilies based on phylogenetic relationship are given as in Figure 1. The black box indicates duplicated gene pairs with domain gains and losses.

Domains are basic functional and structural modules in proteins, and new combination of domains is associated with specific changes in protein functions [27,28]. We analysed the domain architecture of the *SET *gene family in *Populus*. In addition to the SET domain, most of these SET proteins included other domains with known or predicted functions. In particular, each of the six subfamilies found in our analysis had its own specific functional domains (Figure 3), similar to those in *Arabidopsis*, maize and rice [15,16]. In some subfamilies, there were gains and/or losses of domains. PreSET, SET, and PostSET are considered to be primordial domains [29], and they are usually organized in proteins in the order PreSET/SET/PostSET, as seen in the Suv subfamily. Other domains (such as SAR, ZnF_C2H2 and WIYLD) were integrated into this primordial structure to form new gene family members; other members lost one or more of the primordial domains during their evolution (Figure 3). The general patterns of domain gains and losses were similar in the Ash, Trx and E(z) subfamilies.

Some duplicated gene pairs (8/19) also experienced some domain gains and losses (Figure 3, in the black box, and Table 3). For instance, *Suvr5b* gained a ZnF_C2H2 motif near the N-terminus of the encoded protein compared with its counterpart *Suvr5a *(Table 3). Compared with its counterpart *Atxr7a, Atxr7b *lost the Pfam: Luteo_coat domain near the N terminus (Table 3). We have checked all 5181 pairs of genes produced by the most recent rounds of WGD in *Populus*, and found that 783 pairs of them (only 15%) have experienced domain gain and losses. In contrast, among the 18 pairs of WGD duplicates in the SET domain gene family, ~ 45% of them (8 pairs) have experienced domain gain and losses (Fisher's Test, p-value = 0.016). These gains and losses of domains tended to occur near the N terminus (6/8), although they were occasionally found at the C terminus (two pairs: Suvh4b and Suvh4c, Suvr4a and Suvr4b). Most SET domains are located near the C terminus and there might be specific functional constraints that protect the stability of the domain architecture at the C terminus.

**Table 3:** *K_a_/K_s _*and divergence analysis of domain architecture and gene structure of duplicate d *SET *gene pairs in *Populus*

Duplicated pairs		*K_a_/K_s_*	Domain gain/lost			The terminus diversity	
			
			Numbers	Name	Location	5'-terminal	3'-terminal
Suvh2	Suvh9	0.09	/	/	/	1A**,2A,4	4

Suvh3	Suvh10	0.17	/	/	/	3,4	4

Suvh4b	Suvh4c	0.14	1	PreSET	C*	1A,4	1B

Suvh7	Suvh1	0.24	/	/	/	3,4	4

Suvr1	Suvr2	0.35	1	PreSET	N	1A,2A	1A,2A

Suvr4a	Suvr4b	0.23	1	PreSET	C	1A,4	4

Suvr5a	Suvr5b	0.20	1	ZnF-C2H2	N	1A,2A,3,4	4

Atxr5a	Atxr5b	0.11	/	/	/	1A,4	4

Atxr6a	Atxr6b	0.19	/	/	/	1A,2A	/

Ashh2a	Ashh2b	0.22	1	Pfam:zf-CW	N	1A,1B,2A,2B,4	1A,1B,4

Ashh4	Ashh3	0.18	/	/	/	4	4

Atxr3a	Atxr3b	0.28	/	/	/	1A,2A, 4	4

Swna	Swnb	0.26	/	/	/	1A,2A,3,4	4

Clfa	Clfb	0.18	1	SANT	N	1A,1B,2A	4

Atx1	Atx2	0.19	/	/	/	1A,2A,2B,4	4

Atx3a	Atx3b	0.37	1	PWWP	N	1A,2A,4	4

Atx4	Atx5	0.30	/	/	/	1A,2A,4	1B,4

Atxr7a	Atxr7b	0.52	1	Pfam:Luteo_coat	N	1A,2A,4	1B

Setd2a	Setd2b	0.39	/	/	/	1B,3,4	1A

*: N: N-terminus of protein, C: C-terminus of protein. **: Shown in Figure 4

The analysis of the *Populus SET *genes indicated that one of two recent *SET *duplicate undergoes domain gain or loss, during a relatively short period of evolutionary time following a recently WGD event. New domain architectures can drive the evolution of organismal complexity [30]; for example, recombination of domains encoded by genes belonging to the yeast mating pathway had a major influence on phenotype [31]. Therefore, the domain gains and losses in *SET *genes that occurred 13.64-15.93 Mya might have been a strong force of evolution of *Populus *complexity. Because SET proteins are important for histone modification and chromatin structure, they can play crucial roles in regulating gene expression during plant development [6,32]. That their domain architecture has incurred major changes in a short time indicates that epigenetic regulation could be somewhat plastic.

### Expression analysis of *SET *genes in *Populus*

**Figure 5:**
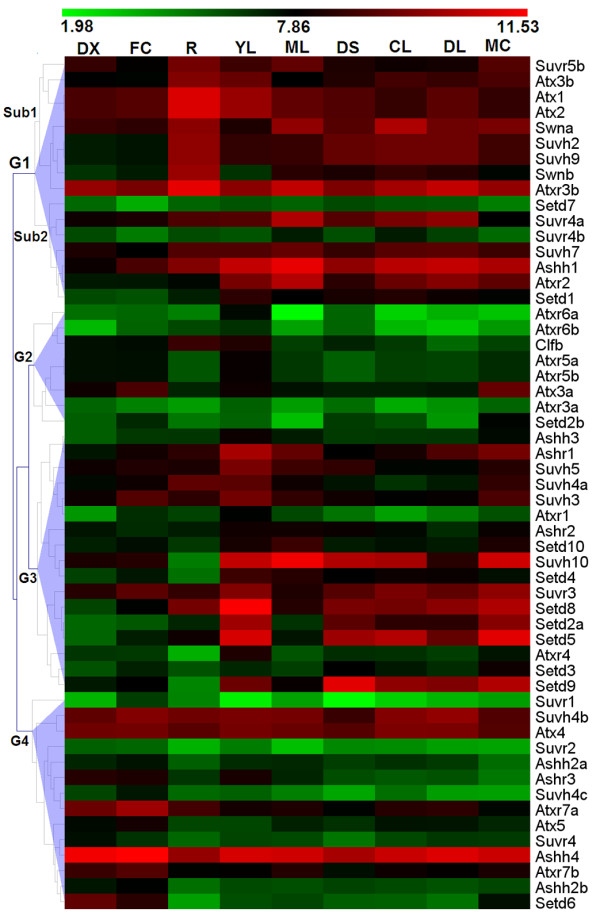
**Hierarchical clustering of expression profiles of *Populus SET *genes across different tissues, organs and treatments**. The expression data were gene-wise normalized and hierarchically clustered based on the Pearson correlation. The relative expression levels of genes in each row are normalized against the maximum value. Colour scale at the top of each dendrogram represents log2 expression values, green represents low levels and red indicates high levels of transcript abundances. CL, continuous light-grown seedling; DL, etiolated dark-grown seedling transferred to light for 3 h; DS, dark-grown seedlings; YL, young leaf; ML, mature leaf; R, root; DX, differentiating xylem; FC, female catkins; MC, male catkins. G1-Sub1, G1-Sub2, G2, G3, G4 represent different subgroup.

To learn about the expression patterns of *SET *genes, we reanalysed the *Populus *microarray data generated by Wilkins and co-workers [33]. Only four *SET *genes (*Suvh1, Atx6, Suvr5a *and *Clfa*) did not have corresponding probes in that dataset, and the expression profiles of the other 55 *SET *genes were analysed as shown in Figure 5 and Additional file 4. We also investigated the frequency of ESTs from EST databases at National Center for Biotechnology Information (NCBI) (March, 2011) and obtained digital expression profiles of 47 *Populus SET *genes; the other 14 *SET *genes did not have EST data (Table 1). The *SET *genes were expressed widely in a number of tissues; intriguingly, expression level of the *SET *genes in specific tissues was higher in young leaves (YL) than in other tissues (t-test, *p*-values < 0.05; Additional file 3: Table S2), except the mature leaves (ML) and roots (R). In contrast, the expression level in differentiating xylem (DX) was lower than in other tissues (t-test, *p*-values < 0.05; Additional file 3: Table S2), except light-grown seedling (CL) and dark-grown seedlings (DS).

Based on hierarchical clustering, the expression patterns of *SET *genes could be divided into four groups: G1-G4 (Figure 5 and Table 4). G1 can be divided into two subgroups: G1-Sub1 and G1-Sub2 (Figure 5). Their expression patterns are listed in Table 4. The ortholog of *Clfb *in *Arabidopsis *was *CLF*, which is required to repress *FLC *[10]. Based on the expression of *Clfb *in *Populus*, we inferred that *Clfb *could have a similar effect on flowering and the shape of mature leaves in *Populus*.

**Table 4:** The expression patterns of *SET *genes

Groups	Members	Expression patterns
G1-Sub1※	Suvr5b/2/9, Swna/b,	All showed high expression level in all tissues except Swnb showed relatively low expression in FC and YL
	
	Atx1/2, Atx3b, Atxr3b	

G1-Sub2	Suvr4a/b, Suvh7, Ashh1,	Most are expressed at lower levels and with different patterns than the first subgroup. Setd7 and Suvr4a have low expression levels in all the tissues.
	
	Atxr2, Setd7/1	

G2	Atxr5a/5b/6a/6b, Atx3a,	Most had very low expression levels in almost all tissues, but the Clfb and Atx3a expressed at higher levels in certain tissues.
	
	Atxr3a, Clfb, Setd2b	

G3	Suvh3/4a/5/10, Suvr3,	Most had high expression levels in YL, ML and others, but low expression in FC. Suvr1, Atxr1 and Ashh3 have low expression levels in all the tissues.
	
	Ashh3, Ashr2/1, Atxr1/4,	
	
	Setd2a/3/4/5/8/9/10	

G4	Ashh2a/2b/4, Ashr3	Most genes had low expression levels in all the tissues, but Ashr3, Atx5, Setd6 and Atxr7b showed high expression levels only in FC and MC
	
	Atxr7a/b, Atx4/5, Setd6	

※Based on hierarchical clustering as shown in Figure 5

**Figure 6:**
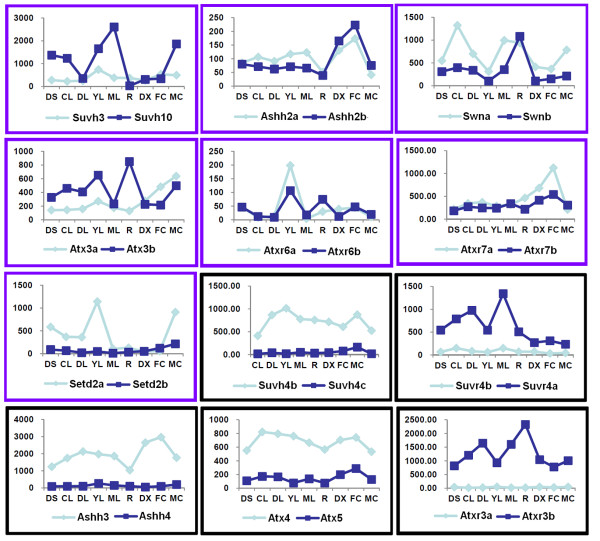
**Two trends of expression patterns of duplicated *SET *gene pairs**. X-axis indicates representative tissues, organs or treatments and Y-axis represents scale. Purple box: both copies of the duplicated pair show complex expression patterns. Black box: one copy of the duplicated pair has low transcription in all tissues compared relative to the other copy.

In addition, we determined the expression profiles of the duplicated *SET *gene pairs. In 12 of the 19 duplicated gene pairs, both copies were co-expressed (values above 100 are considered to be expressed). As shown in Figure 6, the expression profiles of these 12 duplicated *SET *genes formed two patterns: i) one paralog was expressed higher than the other in at least one tissue, whereas the other was expressed at a higher level in some other tissues (Figure 6, purple box); or ii) one paralog had lower expression levels than the other in all tissues (Figure 6, black box).

### Structural divergence of duplicated *Populus SET *gene pairs via four major scenarios

**Figure 4:**
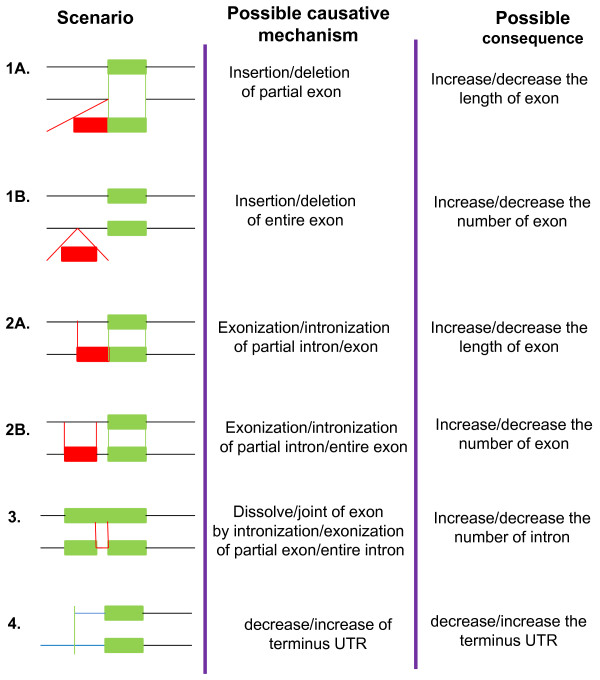
**Scenarios of terminus diversity in duplicated gene pairs of the *SET *gene family in *Populus***. This figure shows potential mechanisms and their possible consequences leading to the four scenarios of terminus diversity observed in duplicated gene pairs. Exons, green filled boxes; introns, black single lines. Untranslated regions (UTRs) are indicated by thick blue lines.

Previous studies showed that structural divergences have played important role during the evolution of duplicates in plants [34,35]. To understand the structural divergence of duplicated *Populus SET *gene pairs, we compared gene structures between the two recent paralogs in each of the 19 duplicated gene pairs. We found that the 3'-and 5'- terminal regions (often containing a portion of the coding region) of most duplicated *Populus SET *pairs were highly divergent (Table 3 and Additional file 5). The 5'-terminal end is more polymorphic than the 3'-terminal end (Mann-Whitney U-test, p-value = 0.00012, Table 3). Further investigation of their sequences revealed that these gene structure changes could have originated from one (or more) of four major scenarios (Figure 4).

We have found that the 3'-terminal and 5'-terminal regions (often with part of the coding region) of most duplicated *Populus SET *gene pairs were highly divergent, particularly the 5' end (Figure 4 and Additional file 5). Further investigation of their sequences revealed that these gene structure differences could have originated from one (or more) of four major scenarios (Figure 4). The first scenario is insertion or deletion (indel) of partial or entire exons (Figure 4-1A and 6-1B). For example, the first exons at the 5' ends of *Atxr7a *and *Atxr7b *matched very well with one other except that the exon in *Atxr7b *lacked three nucleotides at its beginning, indicating an indel event of one codon (Additional file 6 -e.g. 1A). A notable indel case was the loss of an entire exon at the 5' end of the *Clfb *gene in comparison with *Clfa *(Additional file 6 -e.g. 1B). Changes in exon length between most duplicated pairs appeared to have resulted from this scenario (Figure 4-1A &4-1B and Table 3).

The second scenario was the intronization/exonization of a partial or entire exon/intron (Figure 4-2A &4-1B). The second exons at the 5' ends of *Clfa *and *Clfb *were extremely similar in sequence, but *Atxr7a *was about 300 bp longer at its 3' end; we found that part of a *Clfb *intron had been exonized in *Clfa *(Additional file 6 -e.g. 2A). In another interesting case, the second exon in *Ashh2a *matches an intronic region in *Ashh2b*, demonstrating intronization of the whole exon (Additional file 6 -e.g. 2B).

The third scenario was the gain of introns, which divide one exon into two or more smaller ones, or the loss of introns, which unite two or more exons into one longer one (Figure 4-3). The exons near the 5' ends of *Suvh7 *and *Suvh1 *make a persuasive example (Additional file 6 -e.g. 3). There were two exons, 305 and 1657, at the 5' end of *Suvh1*, which flanked a 59 bp intron; whereas there was a single exon, 2010, at the 5' end of *Suvh7*, without an intron.

The last scenario involved an increase or decrease in the UTR at the 5' ends of duplicated pairs genes (Figure 4). Generally, the UTR of one duplicate was longer than the other. For example, *Suvh3 *had a longer UTR at its 5' end than its corresponding region in *Suvh10 *(Additional file 6 -e.g. 4). Taken together, Scenarios 1A and 2A alters exon length, whereas Scenarios 1B and 2B and 3 change exon number, or scenario 3 change intron number. In contrast, the 3' ends of the duplicates were not as different as the 5' ends. The most common case was a change in the length of the UTR at the 3' end.

## Discussion

We have performed systematic phylogenetic analysis of *Populus SET *genes, and determine *K_s _*values of duplicates and examined their expression patterns. Our analyses indicate that *SET *genes have experienced many duplication events during the *Populus *evolutionary history. The relatively recent duplicates are likely the results of whole-genome duplication, and many show structural divergence, including gain/loss of functional domains. Furthermore, at least some recent paralogs exhibit divergent expression patterns. Therefore, *SET *genes in *Populus *provide a case study for evolution of genes following duplication, showing both gene loss and retention with functional divergence at both structural and expression levels.

### Genome duplication and evolution of *SET *genes

There are 59, 47 and 43 known *SET *genes in *Populus, Arabidopsis *and rice, respectively. The *Populus *genome has experienced at least four rounds of genome duplication: two ancient duplication events prior to the gymnosperm-angiosperm divergence and before the diversification of all extent angiosperms, an intermediate event shared with all core eudicots plants, and a recent event occurring after the divergence of the lineages leading to *Populus *and *Arabidopsis *[22,23,25]. Our analysis suggested that, of the 19 duplicated pairs of adjacent *Populus SET *genes on the phylogenetic tree, all resulted from the recent WGD event. Among older duplicates prior to the split of *Populus *and *Arabidopsis*, one or two Suv pairs and one Atx pair likely resulted from the core eudicot WGD, and possibly others from older WGDs. Given that at least four rounds of WGD are known in the *Populus *lineage, many genes loss within the *SET *gene family appears to have occurred in *Populus*. The *Arabidopsis *lineage has experienced two WGD events since its split from the *Populus *lineage and has lost many of the duplicates [36]. Our investigation of *Arabidopsis SET *genes revealed five duplicated gene pairs since its divergence from *Populus*. These five pairs had average *K_s _*values between 0.1817-0.2319, with estimated dates between 27.27-28.78 Mya for the duplication, corresponding to a recent WGD in *Arabidopsis *[22,23]. Furthermore, soybean has experienced one more WGD event than *Populus *since its split with the *Populus *lineage, and it has 25 more *SET *genes than *Populus*. Most of the increased soybean *SET *gene number resulted from the recent WGD (data not shown). Therefore, the expansions in the *SET *gene family in *Arabidopsis, Populus*, and soybean could be explained by gene loss and gain after WGD events.

An increase in the number of regulatory genes (i.e. transcriptional and developmental regulators) is one of important factors that facilitated the evolution of more complex developmental systems [36]. Maere et al. estimated that more than 90% of the increases in *Arabidopsis *regulatory genes were likely caused by genome duplications during the last 150 million years [37]. Our results suggest that WGD could be the main mechanism for the expansion of the *SET *gene family in *Populus*, consistent with this idea. To date, many studies have shown that many members of transcriptional factor (TF) gene families survive after WGD events [33,36,38], but few papers report on epigenetic regulatory gene families. *SET *genes are important epigenetic regulatory gene and have been largely retained after WGDs. Epigenetic regulators modulate expression of a large number of functionally related genes. Therefore, our study suggests that more basic regulatory gene families could have evolutionary mechanisms similar to TF genes, which might contribute to the evolution of gene networks and provide insight into chromatin regulatory evolution.

### Expression profiles of *SET *genes and functional diversity of duplicated pairs in *Populus*

We investigated *Populus *microarray and EST data and found that most *SET *genes are expressed relatively widely, suggesting that the *Populus SET *genes that have survived after WGD events are likely functional. Similarly, almost all *Arabidopsis *and soybean *SET *genes were expressed (Additional file 3: Tables S3, S4), again suggesting that these *SET *genes have functions.

According to the expression patterns of *Populus *genes and the functions of their *Arabidopsis *orthologues, we could hypothesize possible functions of these genes in *Populus*. For example, the orthologue of *Swnb *in *Arabidopsis *is *SWN*, which is involved in H3K27 trimethylation at important floral and shoot developmental genes, including *AGAMOUS *and *SHOOT MERISTEMLESS *(*STM*) [39]. Therefore, *Swnb *might have a function similar to *SWN *in regulating *Populus *flower and shoot development. However, some *SET *genes were specific to *Populus*, for example, *Suvr4a, Suvr4b, Atx3a*, and *Atx3b*. All of these genes have abundant transcripts in *Populus *and are expressed at different levels in different tissues. These results suggest that their counterparts in *Arabidopsis *have been lost and the functions are either not needed in *Arabidopsis *or performed by other genes.

Interestingly, one of the greatest differences between the *Populus SET *genes and *Arabidopsis SET *genes is that there are 19 duplicated *Populus SET *gene pairs but only five *Arabidopsis SET *gene pairs. In 12 of the 19 duplicated gene pairs, both copies of were expressed, with two types of expression patterns.

The first type is that one copy was expressed at higher levels than the other in one or more tissues, but the other copy was higher in some other tissue(s) (Figure 6, purple boxes). For example, *Ashh2a *and *Ashh2b *corresponded to *ASHH2 *in *Arabidopsis*, which negatively regulates shoot branching [40]. The *Ashh2a *and *Ashh2b *duplicates in *Populus *are both expressed at the same stages and could repress shoot branching in *Populus. Swna *and *Swnb *corresponded to the *Arabidopsis SWN*, which exhibits partial functional redundancy with *CLF *and *MEA *[6]. *Populus *is a perennial woody plant with a juvenile-to-mature phase change, so its flowering processes is different from that of *Arabidopsis *and may require two duplicates with somewhat different functions to regulate these processes.

The second type is that one duplicate was expressed at higher levels than the other in all tissues (Figure 6, black boxes), suggesting that ther former has a stronger function than the latter. The *Arabidopsis ATXR3 *gene corresponds to the poplar *Atxr3a *and *Atxr3b. ATXR3 *mutants are smaller with curly leaves, short roots, early flowering, and female sterility [12,13]. The closest *Populus *homologues of *ATXR3 *may also play important roles in regulating broad developmental processes, consistent with one duplicate having wide-ranging high level expression. Similarly, the fact that *Arabidopsis SUVH4 *is responsible for the majority of H3K9 dimethylation in heterochromatin and affects the number of floral organs and the expression of *PHOSPHOANTHRINILATE ISOMERASE *(*PAI*) [6] suggests that the *Populus *homologues *Suvh4a *and *Suvh4b *could also regulate multiple developmental processes.

The two different expression patterns in different tissues of the 12 duplicated gene pairs suggest functional diversification and possible function redundancy (or gene silencing), respectively. Many models for the evolution of gene duplications have been proposed in the past nearly four decades [41]. Recently, divergence in expression between duplicates has also been examined for *Arabidopsis *regulatory genes [42]. The first pattern may support the duplication-degeneration-complementation (DDC) model, in which divergent expression in different tissues allows functional differentiation of the duplicates and finally lead to subfunctionalization or neofunctionalization [43,44]. On the other hand, the latter pattern might suggest functional reduction for one copy (hypofunctionalization), before the weak copy is completely lost (nonefunctionalization) [41,45,46].

## Conclusions

We have shown that *Populus *has gained additional *SET *genes compared with its common ancestor with *Arabidopsis*, due to a WGD since the divergence from the lineage leading to *Arabidopsis*. Those duplicates that have been retained show divergence in both coding regions and expression levels, suggesting that the *SET *genes might have experienced functional diversification, including possible subfunctionalization. The increased number of *SET *genes with potentially distinct functions could have supported the evolution of epigenetic gene regulation of a woody perennial that is more complex than that of an herbaceous annual.

## Methods

### Database search for *SET *genes

The complete genome and predicted proteomes sequence of *Populus *was obtained from the JGI [http://genome.jgi-psf.org/Poptr1_1/Poptr1_1.home.html] database [22]. To identify all genes for proteins containing SET domains in *Populus*, the SET domain PF00856 model from the Pfam database [47,48] was used to perform a local search of the *Populus *predicted proteomes using the HMMER program (2.3.2) [49]. To find genes for similar proteins from unannotated genomic regions, the identified proteins sequences (domains) were used as queries for a gene search against the *Populus *genome sequence using a newly developed software, Phoenix (Protein Homologue Extraction, Sun et al., unpublished). An *e*-value cutoff value of 1e-5 was used for further analysis. *SET *genes from *Arabidopsis *were obtained as described by Zhang et al. [15].

### Multiple sequence alignment, gene structure and protein architecture analyses

Multiple sequence alignment using MUSCLE with default parameters [50] was performed on SET proteins, and the alignment was subsequently adjusted manually. Gene structure information, including the intron/exon distribution pattern, was obtained by GSDS (Gene Structure Display Server), developed by CBI [51]. Structural motif annotation was performed using the SMART [http://smart.embl-heidelberg.de] and Pfam [http://pfam.sanger.ac.uk] databases.

### Genome localization and syntenic analysis of *SET *genes

Information on chromosomal location was gathered from the *Populus *genome browser. MapInspect [http://www.plantbreeding.wur.nl/UK/software_mapinspect.html] was used to map the *SET *genes onto chromosomes. Syntenic information was collected from the Plant Genome Duplication Database [PGDD: http://chibba.agtec.uga.edu/duplication] [23,26].

### Calculating *K_s _*and dating the duplication event

Protein amino acid sequences of all the duplicated gene pairs from *Populus *were aligned using MUSCLE with default parameters [50], and the results were used to guide the alignments of DNA coding sequences (CDS) by Pal2Nal [52]. *K_s _*and *K_a_*, the number of synonymous and nonsynonymous substitutions per site, were determined using the aligned CDS by yn00 in PAML 4.3 [53].

In dating segmental duplication events, the approximate date of the duplication event was calculated using the mean *K_s _*values from T = *K_s_*/2λ, where the clock-like rate (λ) for *Populus *was 9.1 × 10^-9 ^[54]. Similarly, the average *K_s _*of all the duplicated *SET *gene pairs from *Populus *and *Arabidopsis *was calculated by the same method, where the clock-like rate (λ) for *Arabidopsis *was 1.5 × 10^-8^.

### Tree building

Phylogenetic trees for the aligned SET protein sequences were constructed using NJ, ML, and BI methods. The NJ tree was constructed using MEGA [55] with the "pairwise deletion" option and "Poisson correction" model. Bootstrap support was estimated from 1000 replicates to evaluate the reliability of internal branches. ML trees were generated using PhyML version 3.0.1, with 100 nonparametric bootstrap replicates and WAG model [56]. MrBayes software [57] was used to construct BI trees, using the WAG model of evolution, after running for 1,000,000 generations, with four Markov chains sampled every 1000 generations.

### Gene expression analysis

To search for *SET *genes from *Populus *ESTs, we used the *Populus SET *CDS as query sequences to search for highly similar ESTs sequences (at least 160 bp and 95% identity) in *Populus *using the NCBI database. We also analyzed the public microarray dataset of *Populus *[33]; we normalized and hierarchically clustered them based on Pearson coefficients with average linkage in the MeV (version 4.8) program [58]. Furthermore, we also got the RNA-seq dataset of soybean [59].

## Abbreviations

K: Lysine; ML: Maximum likelihood; BI: Bayesian inference; NJ: Neighbour-Joining; Mya: Million years ago; WGD: Whole-genome duplication; PGDD: Plant genome duplication database; TF: Transcriptional factor; EST: Expressed sequence tag; NCBI: National center for biotechnology Information; UTR: Untranslated region; *K_s_*: Synonymous substitutions rate: *K_a_*: Non-synonymous substitution rate.

## Authors' contributions

LSZ and LL conceived the study and performed the analyses. LL and LSZ wrote the manuscript. HM provided support for the study, contributed to the discussion and revised the manuscript. SLZ revised the manuscript and contributed to discussion. All authors read and approved the final manuscript.

## Supplementary Material

Additional file 1 **Unrooted phylogenetic trees constructed using (A) ML and (B) BI methods based on SET amino acid sequences from *Populus *and *Arabidopsis *SET proteins**. Click here for file

Additional file 2 **Detailed locations of all duplicated pairs of *SET *genes in *Populus *from recent and ancient polyploidy events in the syntenic region**. Click here for file

Additional file 3 **Table-S1 *K_s _*of *SET *gene pairs in Suv, Ash, Atxr5, E(z) and Trx subfamilies**. Table-S2 Raw data from microarray expression analyses for *Populus *SET genes. Sample abbreviations are defined in Figure 6 and Additional file 4. Table-S3 Raw data from ESTs for *Arabidopsis SET *genes. Table-S4 Raw data from transcriptome data for soybean *SET *genes. Click here for file

Additional file 4 **Transcript abundance in different subgroups across different tissues, organs, and treatments in *Populus *based on the genome-wide microarray data**. Sample abbreviations are defined in Figure 6 Click here for file

Additional file 5 **Structure of duplicated gene pairs within the *Populus SET *gene family**. Exons, green filled boxes; introns, black single lines. UTRs are indicated by thick blue lines at both ends. Intron phases 0, 1 and 2 are indicated by numbers 0, 1 and 2 in the figure. Click here for file

Additional file 6 **Examples of five scenarios for terminus diversity in duplicated gene pairs of the *SET *family in *Populus***. This number corresponds to the one in Figure 6, and the red box represents the location of the mutations causing the patterns. Click here for file
