# Supplementary figures and images for: Expansion and diversification of the SET domain gene family following whole-genome duplications in Populus trichocarpa
Source: BMC Evol Biol. 2012 Apr 12;12:51. doi: 10.1186/1471-2148-12-51 (PMC3402991; doi:10.1186/1471-2148-12-51)

ML

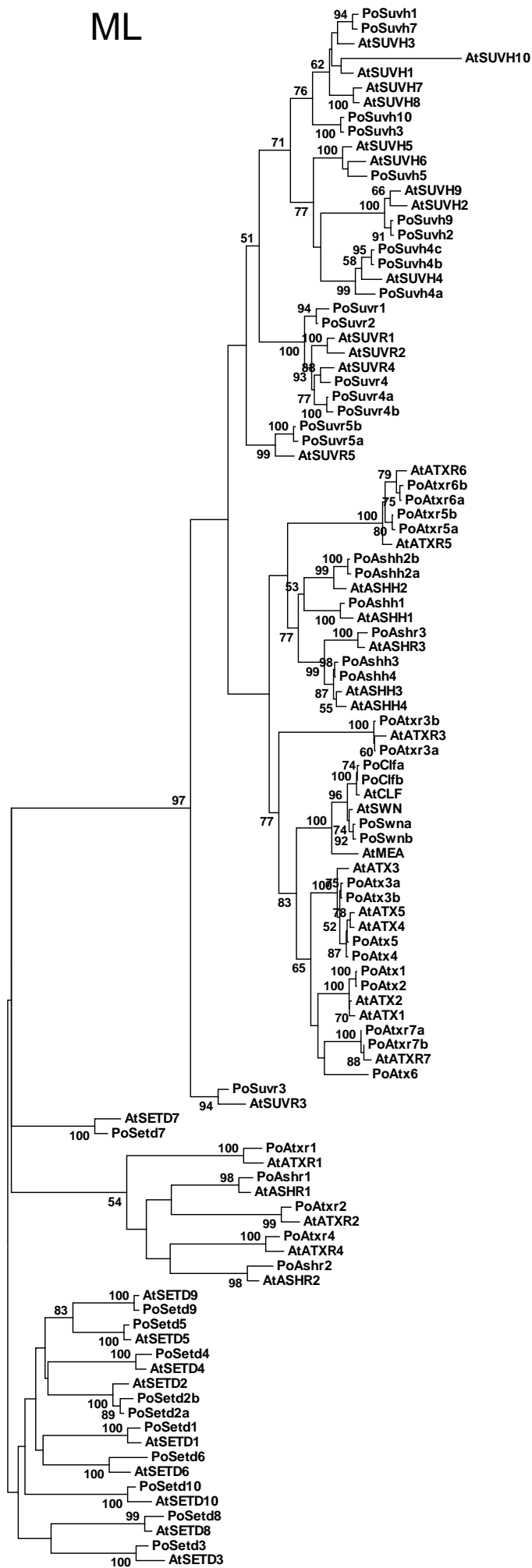

Bayes

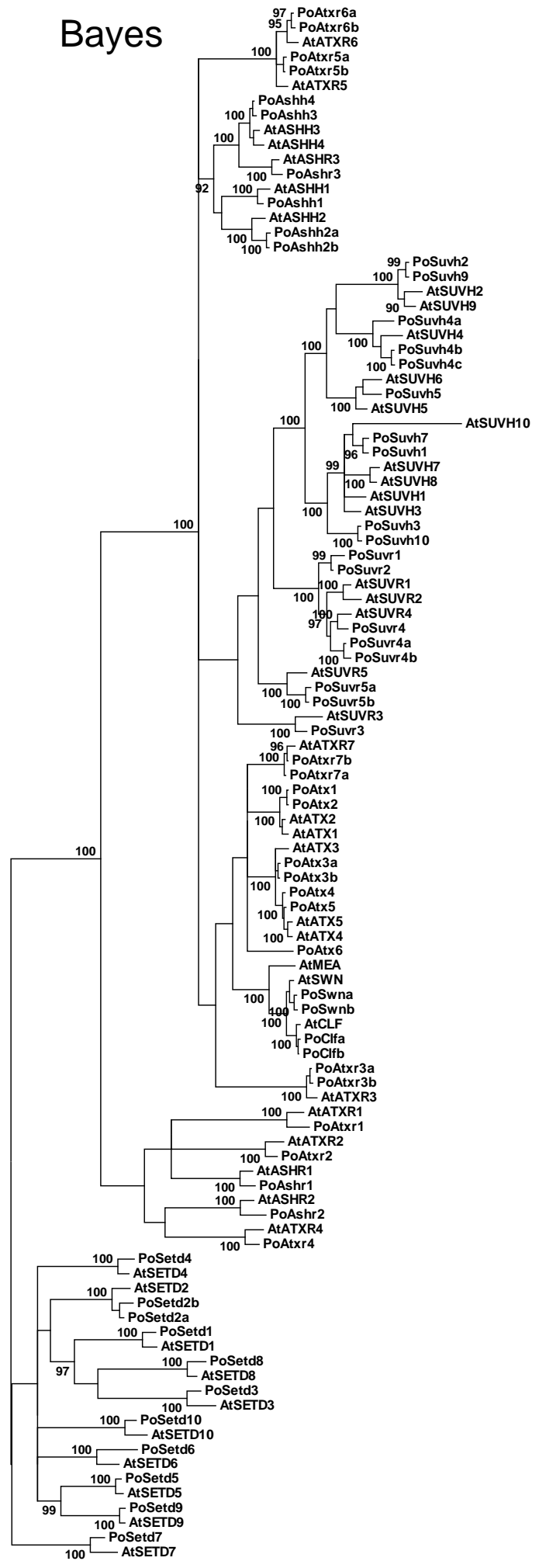

Supplement: Additional file 1 — Unrooted phylogenetic trees constructed using (A) ML and (B) BI methods based on SET amino acid sequences from Populus and Arabidopsis SET proteins. [file 1471-2148-12-51-S1.PDF]

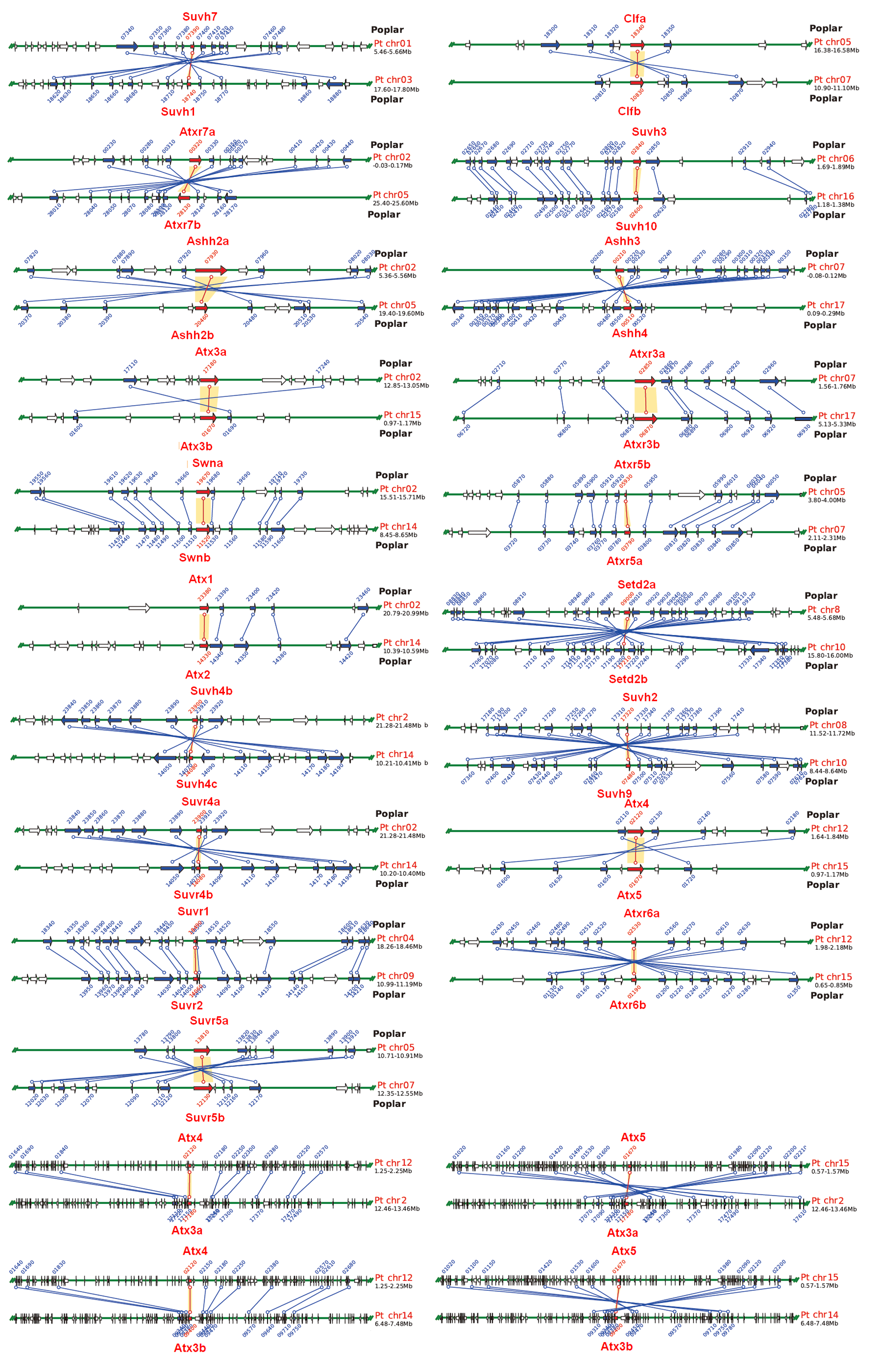

Supplement: Additional file 2 — Detailed locations of all duplicated pairs of SET genes in Populus from recent and ancient polyploidy events in the syntenic region. [file 1471-2148-12-51-S2.TIFF]

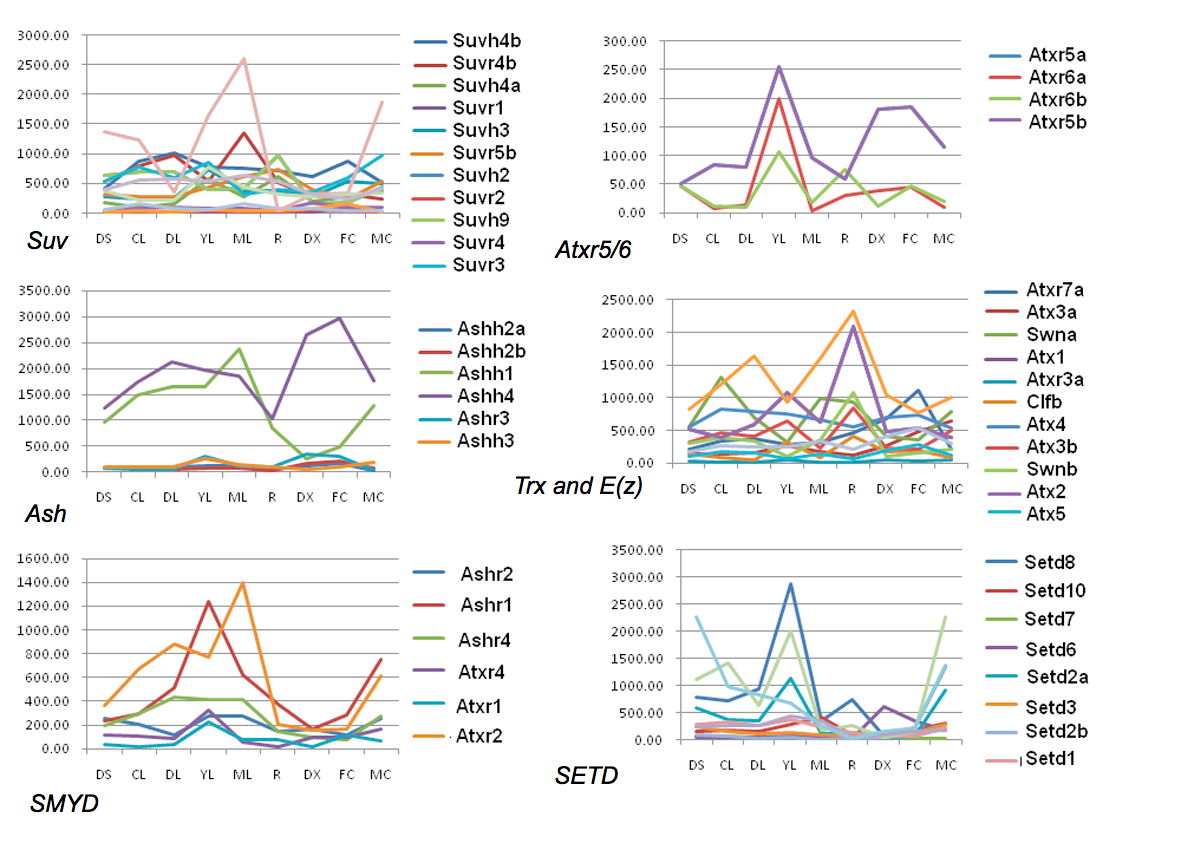

Supplement: Additional file 4 — Transcript abundance in different subgroups across different tissues, organs, and treatments in Populus based on the genome-wide microarray data. Sample abbreviations are defined in Figure 6 [file 1471-2148-12-51-S4.TIFF]

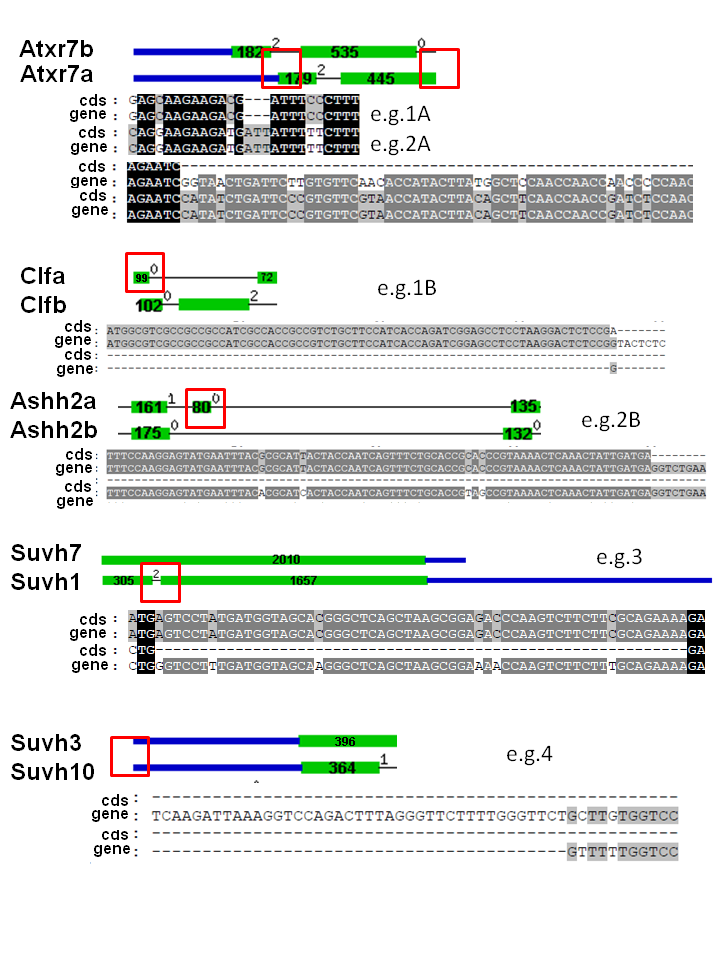

Supplement: Additional file 6 — Examples of five scenarios for terminus diversity in duplicated gene pairs of the SET family in Populus. This number corresponds to the one in Figure 6, and the red box represents the location of the mutations causing the patterns. [file 1471-2148-12-51-S6.TIFF]
